# Inhibitory effect of ficin on *Candida albicans* biofilm formation and pre-formed biofilms

**DOI:** 10.1186/s12903-022-02384-y

**Published:** 2022-08-13

**Authors:** Jiantao Yu, Fan Wang, Yan Shen, Fangzheng Yu, Lili Qiu, Lingjun Zhang, Yanhan Chen, Qing Yuan, Huan Zhang, Yan Sun, Keke Zhang

**Affiliations:** 1 School and Hospital of Stomatology, Wenzhou Medical University, 325027 Wenzhou, People’s Republic of China; 2 Institute of Stomatology, School and Hospital of Stomatology, Wenzhou Medical University, Wenzhou, 325027 People’s Republic of China

**Keywords:** *Candida albicans*, Ficin, Biofilm formation, Pre-formed biofilm

## Abstract

**Background:**

To investigate the effect of ficin, a type of proteases, on *Candida albicans* (*C. albicans*) biofilm, including forming and pre-formed biofilms.

**Methods:**

Crystal violet tests together with colony forming unit (CFU) counts were used to detect fungal biofilm biomass. Live/dead staining of biofilms observed by confocal laser scanning microscopy was used to monitor fungal activity. Finally, gene expression of *C. albicans* within biofilms was assessed by qRT-PCR.

**Results:**

According to our results, biofilm biomass was dramatically reduced by ficin in both biofilm formation and pre-formed biofilms, as revealed by the crystal violet assay and CFU count (*p* < 0.05). Fungal activity in biofilm formation and pre-formed biofilms was not significantly influenced by ficin according to live/dead staining. Fungal polymorphism and biofilm associated gene expression were influenced by ficin, especially in groups with prominent antibiofilm effects.

**Conclusions:**

In summary, ficin effectively inhibited *C. albicans* biofilm formation and detached its preformed biofilm, and it might be used to treat *C. albicans* biofilm associated problems.

## Introduction

Fungal infections are usually difficult to diagnose, with delayed diagnosis, and efficacious antifungal strategies are lacking [1]. *Candida albicans* (*C. albicans*) is the most familiar opportunistic pathogen and is regarded as the foremost cause of invasive candidiasis. Infection of this fungus can be transmitted from the mucosa to the bloodstream, and is especially severe in immunocompromised people, such as AIDS patients [2, 3]. As an opportunistic oral fungal pathogen, *C. albicans* has been reported to be closely related to denture stomatitis and has been used in several protocols to construct an animal model of denture stomatitis [4, 5]. In addition, *C. albicans* prevalence shows a positive correlation with severity of early childhood caries, and a synergic relationship between this fungus and opportunistic cariogenic *Streptococcus mutans* has been gradually revealed [6]. What’s more, *C. albicans* colonization may be related to peri-implant infections in the oral cavity [7].

Most diseases caused by *C. albicans* are associated with its biofilm. Progressive *C. albicans* biofilms, once formed, can provide protection to the fungi residing within it, thus making *C. albicans* resistant to most antifungal drugs, including fluconazole and amphotericin B, which are commonly used [8]. *C. albicans* within biofilms is 1000 times more resistant to antifungal agent than planktonic cells [9]. Antifungal drug resistance mechanisms of *C. albicans* biofilms include extracellular matrix, persister cells, enhanced drug efflux pumps, enhancive cell density, stress response while depressed metabolic activity [2, 10]. Moreover, commonly used antifungal agents have facilitated the appearance and dissemination of drug resistant *C. albicans* such as fluconazole-resistant clinical isolates [11, 12]. Therefore, a new strategy to control *C. albicans* biofilms is urgent needed to manage *C. albicans* biofilm associated diseases especially in the so called post-antibiotic era.

Enzymatic degradation of biofilms has been proposed as an alternative strategy due to superiority of rare resistance development [13]. Ficin is a sulfhydryl proteases with inherent peroxidase-like activity [14]. The antibiofilm effect of ficin was first reported in *Staphylococcus aureus* (*S. aureus*) together with *Staphylococcus epidermidis* (*S. epidermidis*), and these two kinds of biofilms were effectively destroyed by this protease [15]. When ficin is immobilized in chitosan, it also shows anti-biofilm and wound-healing activity [16]. Our previous study displayed that ficin not only significantly inhibits biofilm formation of opportunistic cariogenic *Streptococcus mutans* (*S. mutans*), but also suppresses its cariogenic virulence including acid production and EPS synthesis [17]. Most recently, ficin was reported to have effectivity against *Salmonella Enterica* serovar Thompson biofilms [18]. However, the effect of ficin on fungal biofilms remains unknown. Therefore, in this study, we evaluated the ficin’s anti-biofilm characteristics of ficin against *C. albicans* biofilm to evaluate its potential to control *C. albicans* biofilms.

## Materials and methods

### Fungi and culture conditions

*C. albicans* strain SC5314 used in this experiment (Institute of Stomatology, School and Hospital of Stomatology, Wenzhou Medical University). Briefly, a single clone grown on Sabouraud’s agar plates (SDA; Solarbio Science& Technology Co., Ltd., China) was cultured overnight for proliferation in yeast peptone dextrose broth (YPD, Solarbio Science & Technology Co., Ltd., Beijing, China) at 37 °C under aerobic conditions.

A total of 5 × 10^5^ CFU/mL of overnight cultured *C. albicans* was inoculated in morpholinepropanesulfonic acid (MOPS, Solarbio Science & Technology Co., Ltd., Beijing, China) modified RPMI-1640 media (Gibco, Bethesda, MD, USA) with different concentrations of ficin, followed by 48 h of biofilm formation. For pre-formed biofilm, after 48 h of biofilm formation without ficin, the culture media was replaced by MOPS modified RPMI-1640 media supplemented with different ficin contents for another 48 h. Media without ficin was set as a blank control and 80 μM fluconazole served as a positive control [8].

### Crystal violet assay

Biofilms in 96-well platez (200 μL culture volume) were fixed with methanol, and stained for 30 min by 0.1% (w/v) crystal violet. The dyed biofilms were observed and photographed using a stereomicroscope (Nikon SMZ800N, Nikon Corporation, Japan). Then, 150 μL of 33% acetic acid solution was added to elute the crystal violet stain from the biofilms. The eluent was transferred to another 96-well plates, and the OD at 590 nm was recorded by a microplate reader (SpectraMaxM5, Molecular Devices, USA) [19].

### Colony forming unit (CFU) counts

Biofilms in 96-well plates (200 μL culture volume) were collected in PBS and sonicated/vortexed completely. After gradient dilution with PBS, 100 μL of fungal suspensions was spread onto SDA solid medium and cultured for 48 h at 37 °C aerobically to support fungal growth. The clones grown on medium were counted [20].

### Live/dead staining and CLSM imaging

Heat-polymerized acrylic resin (Jianchi Dental Equipment, Changzhi, China) was used to support *C. albicans* in this test as previously described [20]. Specimens were cut into 1 cm squares that were 2 mm thick, polished and sterilized by ethylene oxide.

Biofilms in 24-well plates (2 mL culture volume) were dyed by LIVE/DEAD® BacLight™ Bacterial Viability Kits (Thermo Fisher Scientific, Waltham, MA, USA) according to the product manual. Both SYTO 9 and propidium iodide were used to stain live and dead *C. albicans* for 30 min, respectively. The stained biofilms were randomly captured with a 60 × objective lens by CLSM (Nikon A1, Nikon Corporation, Japan). The live fungal ratio was analyzed according to fungal coverage with Image Pro Plus 6.0 software (Media Cybernetics, Inc., Silver Spring, MD, USA) based on 5 random pictures in each group.

### RNA isolation and qRT-PCR

*C. albicans* biofilms in 96-well plate (200 μL culture volume) were collected, and total RNA was isolated by a TRIzol dependent method [8]. Then quality testing of RNA was conducted by Nanodrop 2000 spectrophotometer (Fisher Scientific, Pittsburg, PA, USA) and electrophoresis. Then reverse transcription was presented using a PrimeScript™ RT reagent Kit with gDNA Eraser (Takara Bio Inc., Otsu, Japan) following the manufacturer's instructions. The qRT-PCR was carried out with TB Green® Premix Ex Taq™ II (Tli RNaseH Plus, Takara Bio Inc., Otsu, Japan), and the reaction volume was 20 μL (primers are listed in Table 1). PCR procedure (95 °C for 30 s, and 35 cycles including 95 °C for 5 s, 55 °C for 30 s, 72 °C for 30 s) was run in a Step One Plus Real-Time PCR System (Applied Biosystems, CA, USA), and gene expression was normalized by the 2^−ΔΔCT^ method.

**Table 1:** Primers used in this study

Primers	Nucleotide sequence (5’-3’)	References
*18S*-f	CACGACGGAGTTTCACAAGA	[21]
*18S*-r	CGATGGAAGTTTGAGGCAAT	
*hwp1*-f	GCTCCTGCTCCTGAAATGAC	[21]
*hwp1*-r	CTGGAGCAATTGGTGAGGTT	
*ywp1*-f	GCTACTGCTACTGGTGCTA	[21]
*ywp1*-r	AACGGTGGTTTCTTGAC	
*als1*-f	GACTAGTGAACCAACAAATACCAGA	[22]
*als1*-r	CCAGAAGAAACAGCAGGTGA	
*als3*-f	CAACTTGGGTTATTGAAACAAAAACA	[21]
*als3*-r	AGAAACAGAAACCCAAGAACAACC	
*bgl2*-f	ATGGGTGATTTGGCTTTCAA	[23]
*bgl2*-r	CAGCTGGACCAAGGTTTTGT	

### Statistical analysis

All tests were repeated at least three times independently. All data are presented as the mean ± standard deviation. One-way analysis of variance (ANOVA) and Tukey’s multiple comparison tests were used to analyze statistical significance (*p* < 0.05) using SPSS software 16.0 (SPSS Inc., Chicago, IL, USA).

## Results

### Fungal biofilm formation and pre-formed biofilms were suppressed by ficin, as revealed by the crystal violet assay

**Fig. 1:**
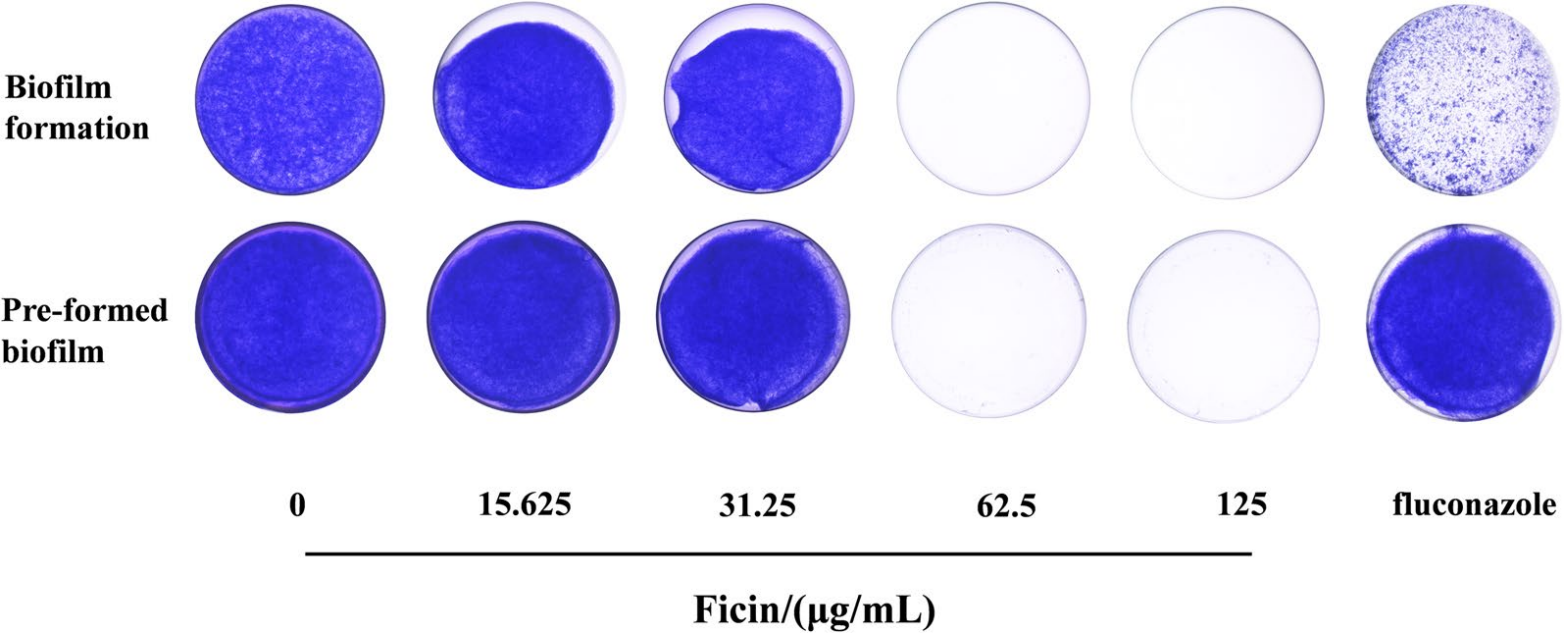
Crystal violet stained biofilms

**Fig. 2:**
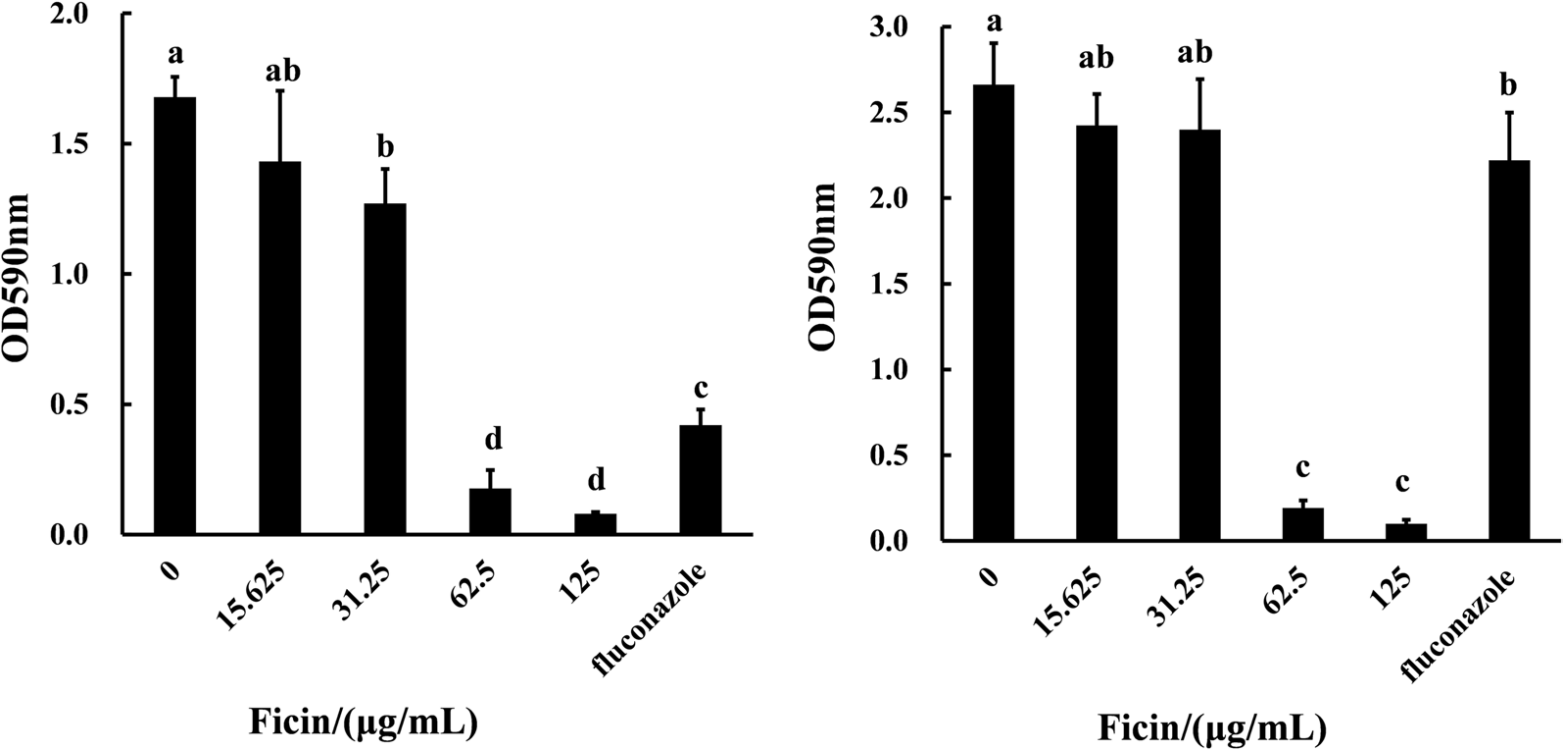
Quantitative analysis of crystal violet stained biofilm. **A** OD values of 48 h *C. albicans* biofilm (biofilm formation). **B** OD values of 96 h *C. albicans* biofilm (pre-formed biofilm). Different letters indicate statistically significant differences

Images of crystal violet stained biofilms showed that 15.625 and 31.25 μg/mL ficin had limited effects on biofilm formation and pre-formed biofilms of *C. albicans* (Fig. 1). Treatment with 62.5 and 125 μg/mL ficin not only inhibited *C. albicans* biofilm formation, but also significantly suppressed pre-formed biofilms (Fig. 1). Little biofilm was detected in these two concentrations. Fluconazole, a positive control, significantly suppressed biofilm formation but had little effect on pre-formed biofilm (Fig. 1). Quantitative results were similar, with 62.5 and 125 μg/mL ficin prominently reducing the OD (Fig. 2).

### Ficin decreased the CFU of C. albicans biofilm

**Fig. 3:**
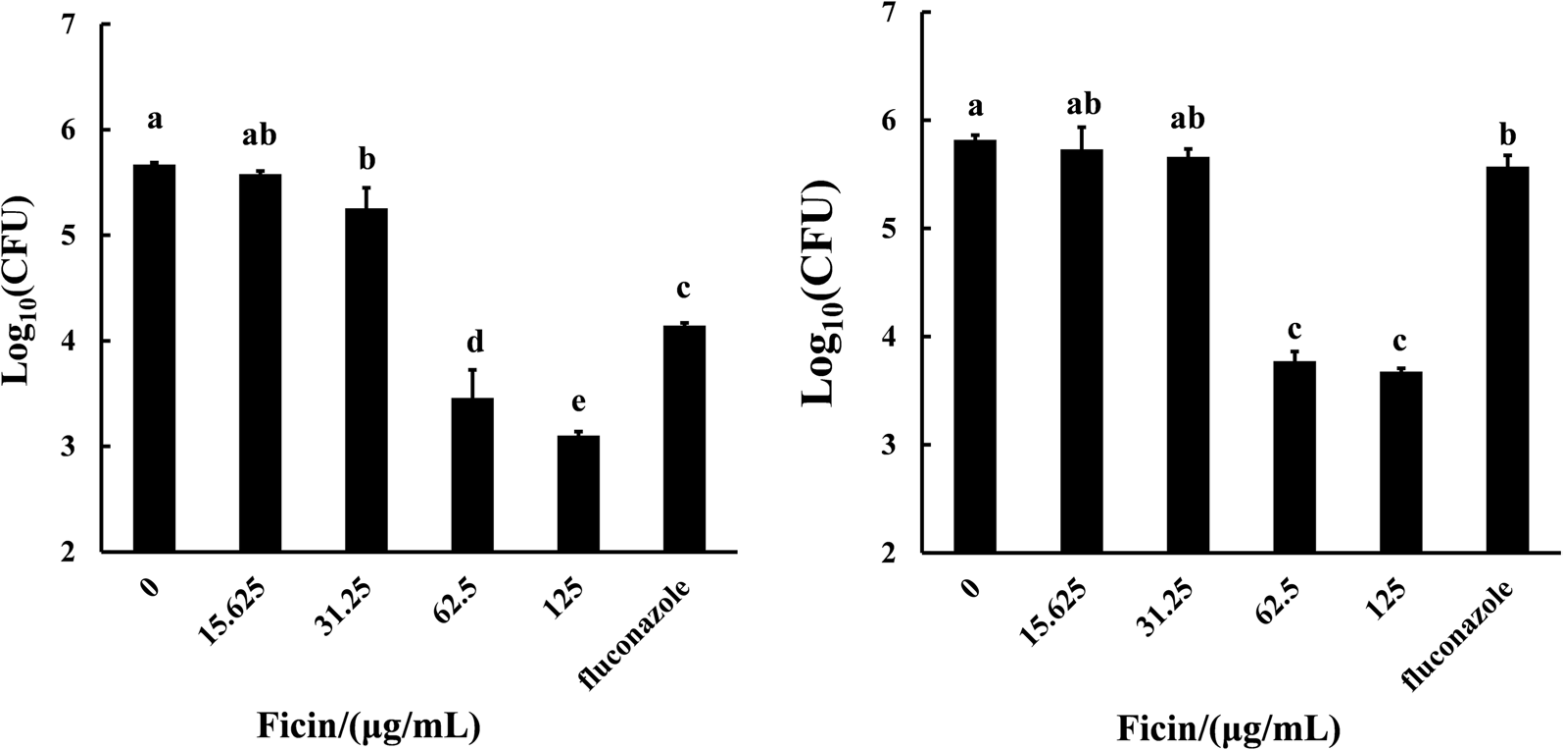
CFU of *C. albicans.* **A** 48 h *C. albicans* biofilm (biofilm formation). **B** 96 h *C. albicans* biofilm (pre-formed biofilms). Different letters indicate statistically significant differences

Ficin decreased the CFU of *C. albicans* both in biofilm formation and pre-formed biofilms (Fig. 3). During biofilm formation, 62.5 and 125 μg/mL ficin and fluconazole caused reduction of 2.57, 2.21 and 1.53 log_10_(CFU) respectively (Fig. 3A, *p* < 0.05). For pre-formed biofilm, fluconazole only led to 0.25 log_10_(CFU) decrease, which revealed a limited effect (Fig. 3B). However, 62.5 and 125 μg/mL ficin caused decreases of 2.14 and 2.05 log_10_(CFU) (Fig. 3B, *p*  < 0.05).

### Ficin did not change fungal activity within biofilms

**Fig. 4:**
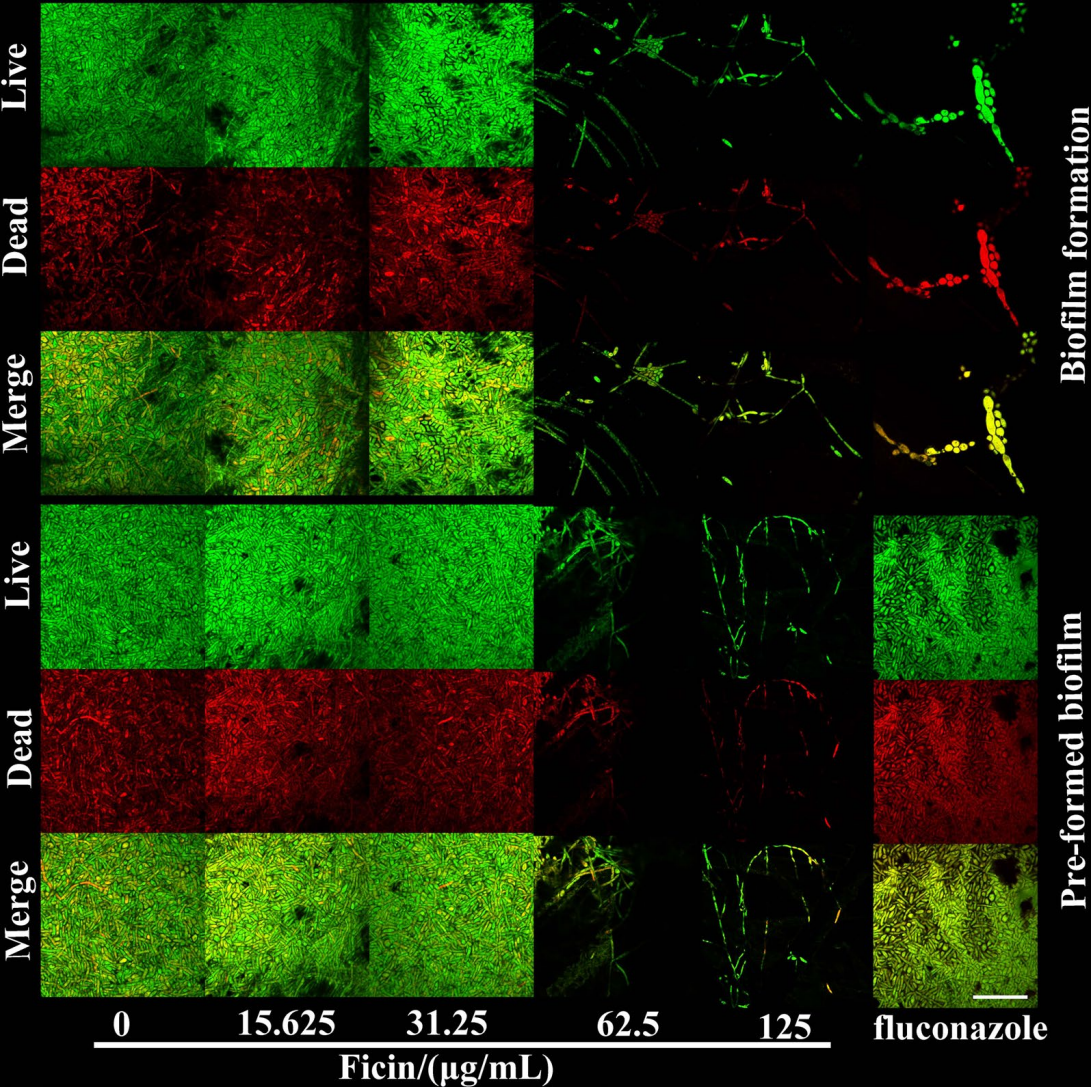
Live/dead staining of *C. albicans* biofilm. Live *C. albicans* stained green, dead *C. albicans* stained red, scale bar = 50 μm

**Fig. 5:**
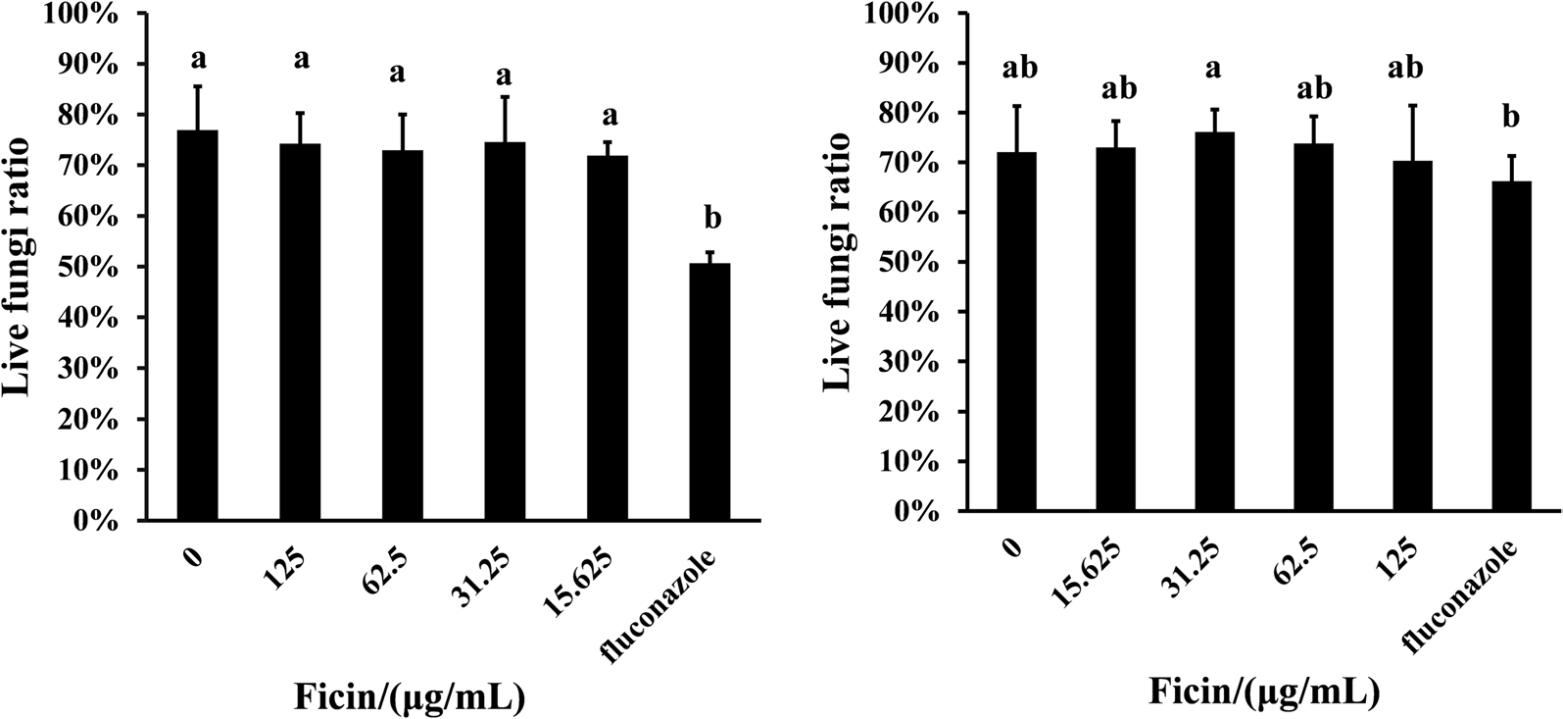
Live *C. albicans* within biofilms. **A** Live fungi ratio in 48 h *C. albicans* biofilm (biofilm formation). **B** Live fungi ratio in 96 h *C. albicans* biofilm (pre-formed biofilms). Different letters indicate statistically significant differences

According to live/dead staining results, ficin did not significantly change fungal activity within biofilm formation and pre-formed biofilms (Figs. 4 and 5). Although 62.5 and 125 μg/mL ficin inhibited and detached biofilms, respectively, it did not prominently influence fungal activity. Fluconazole seemed to affect biofilm activity in biofilm formation and had a limited effect on pre-formed biofilms (Figs. 4 and 5).

### Ficin affected gene expression of C. albicans within two biofilm associated processes

**Fig. 6:**
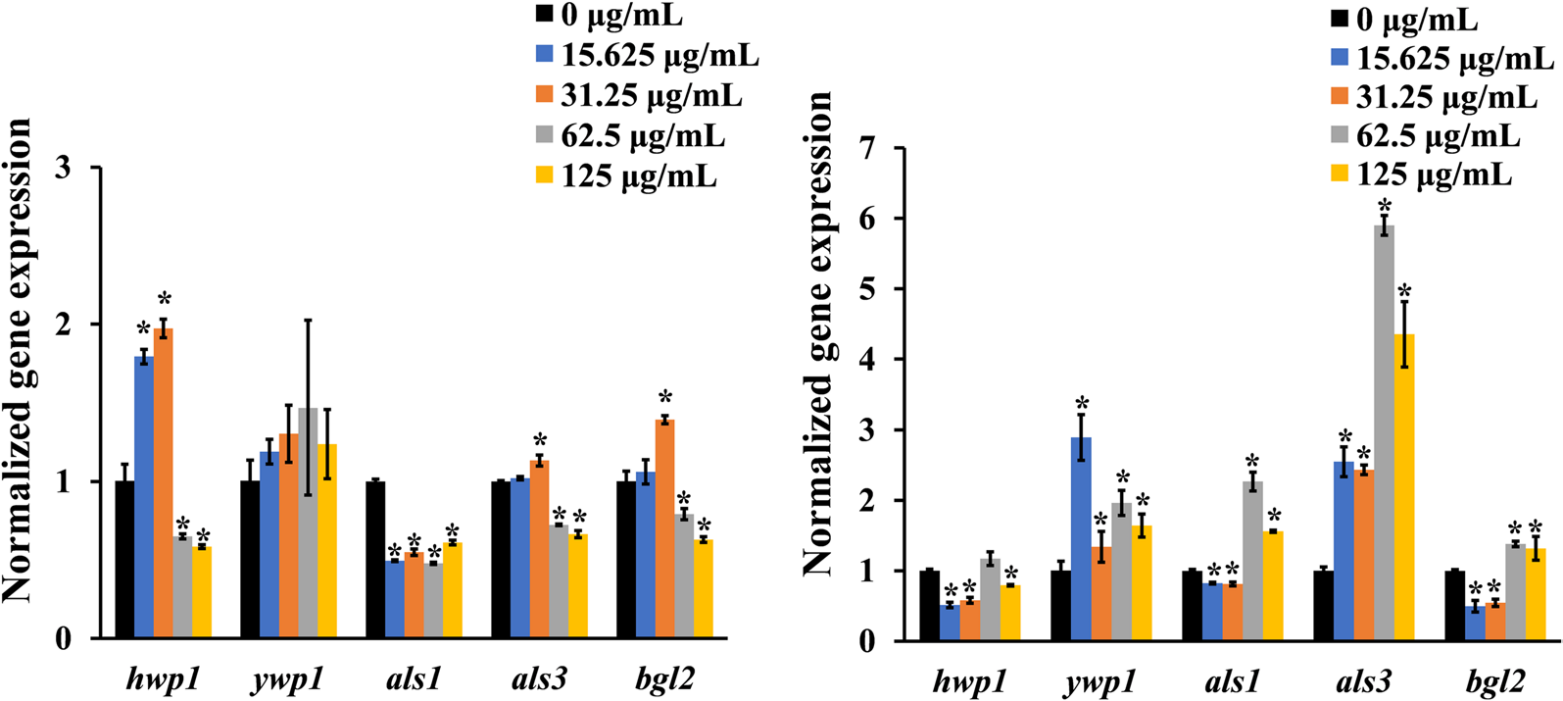
Gene expression of *C. albicans* in biofilms. **A** Gene expression of fungi in 48 h *C. albicans* biofilm (biofilm formation). **B** Gene expression of fungi in 96 h *C. albicans* biofilm (pre-formed biofilms). * indicated statistically significant differences when compared with control

During *C. albicans* biofilm formation, expression of most gene including *hwp1*, *als1*, *als3*, and *bgl2* was suppressed significantly in the 62.5 and 125 μg/mL groups (*p* < 0.05); however, *ywp1* was upregulated but not significantly (Fig. 6A, *p*  > 0.05). In the 15.625 and 31.25 μg/mL groups, *hwp1*, *als3* and *bgl2* were upregulated, but *als1* was downregulated (Fig. 6A, *p*  < 0.05). In pre-formed biofilms, *ywp1* and *als3* were upregulated, whereas *hwp1* (except 62.5 μg/mL) was downregulated significantly in all ficin groups (Fig. 6B, *p*  < 0.05). *hwp1*, *als1* and *bgl2* expression was inhibited in the 15.625 and 31.25 μg/mL groups (Fig. 6B, *p*  < 0.05). In the 62.5 and 125 μg/mL group, *als1* and *bgl2* were upregulated (Fig. 6B, *p*  < 0.05).

## Discussion

In this study, we explored the effect of ficin on *C. albicans* biofilms. Our results showed that ficin not only inhibits *C. albicans* biofilm formation, but also detaches pre-formed biofilms, which for the first time indicates its anti-fungal biofilm effect. Previous studies have confirmed that ficin controls bacterial biofilms, including those of *S. aureus*, *S. epidermidis*, *S. mutans* and *Salmonella Enterica* [15–18]*.* Combined with the findings of this study, we conclude that ficin controls not only bacterial biofilms but also fungal biofilms. Pre-formed biofilms show stronger resistance to stress than biofilm formation [24]. Therefore, studies have reported that antibiofilm agents, including the antifungal fluconazole, inhibit biofilm formation but do not suppress pre-formed biofilms [24–26]. The effectiveness of ficin on both biofilm formation and pre-formed biofilm reveals its advantage over fluconazole to some extent, except for the preponderance of enzymatic degradation to control biofilms, rare resistance [13].

The antibiofilm mechanism of ficin against *C. albicans* in this study is unknown. Our data show that ficin barely influences fungal activity within biofilms, as disclosed by biofilm live/dead staining, which was consistent with previous studies [15, 17]. In *S. aureus* and *S. epidermidis* biofilms, matrix proteins are hydrolyzed by ficin without germicidal effects [15]. For biofilm formation of *S. mutans*, ficin reduced total biofilm proteins and decreased the molecular weight of isolated extracellular proteins, but did not affect bacterial growth and activity [17]. The extracellular matrix plays a vital role in mature *C. albicans* biofilm structures, in which the most abundant components are proteins (approximately 55%) [27]. Because it is a protease, the anti-biofilm effect of ficin might occur through degradation of extracellular proteins. In addition, as ficin showed an anti-biofilm effect without a fungicidal effect, to eradicate biofilms thoroughly, combination therapy that combines ficin with a fungicidal agent without antagonistic action might be a good choice, enabling ficin to inhibit and detach biofilms and fungicidal agents to eliminate nonbiofilm cells simultaneously [15].

Polymorphism is important for the pathogenicity of *C. albicans*. The hyphal form is more invasive, whereas the yeast form is related to dissemination [28]. This might partly explain why the yeast form associated gene *ywp1* tended to upregulated but the hypha formation related gene *hwp1* was suppressed at ficin concentrations that both inhibited biofilm formation and detached pre-formed biofilms significantly. Biofilm associated genes, including adhesion *als1*, *als3* and *bgl2*, which encode β-glucans, were repressed during the biofilm formation process, whereas t they were upregulated in preformed biofilms under marked antibiofilm ficin concentration. One possibility is that *C. albicans* within pre-formed biofilm upregulates those biofilm genes to attempt to maintain its biofilm form and that *C. albicans* barely formes biofilms at those concentrations, thus downregulating expression of *als1*, *als3* and *bgl2* in preparation for diffusion to another hospitable environment in the biofilm formation process.

One limitation of the present study is that a biofilm model involving one species was used. In nature, biofilms always exist in mixed-species, including *C. albicans* associated infections [29, 30]. Multi-species biofilms show more resistance than single species biofilms [31, 32]. In addition, the virulence and pathogenicity of *C. albicans* are enhanced in biofilms containing oral bacteria [33]. Though ficin showed a predominant anti-*C. albicans* biofilm effect at a safe concentration in this study, complex *C. albicans* involved biofilm models or in situ *C. albicans* containing biofilm models should be used to further evaluate the anti-biofilm effect of ficin [17]. Furthermore, in vivo experiments are encouraged to assess antifungal biofilm effect of ficin. Moreover, modifying materials with ficin to obtain antibiofilm characteristics is a research direction for the future.

## Conclusions

Ficin exhibits an inhibitory effect against *C. albicans* biofilm, and it might has potential in the management of *C. albicans* biofilm associated problems.

## Data Availability

Data are available from the corresponding author on reasonable request.
